# ELF4 is critical to zygotic gene activation and epigenetic reprogramming during early embryonic development in pigs

**DOI:** 10.3389/fvets.2022.954601

**Published:** 2022-07-19

**Authors:** Lijing Shi, Yanhui Zhai, Yuanshen Zhao, Xiangjie Kong, Daoyu Zhang, Hao Yu, Ziyi Li

**Affiliations:** ^1^Key Laboratory of Organ Regeneration and Transplantation of Ministry of Education, First Hospital, Jilin University, Changchun, China; ^2^College of Animal Science, Jilin University, Changchun, China

**Keywords:** ELF4, DNA damage, H3K9me3, DNA methylation, pigs, zygotic gene activation

## Abstract

Zygotic gene activation (ZGA) and epigenetic reprogramming are critical in early embryonic development in mammals, and transcription factors are involved in regulating these events. However, the effects of ELF4 on porcine embryonic development remain unclear. In this study, the expression of ELF4 was detected in early porcine embryos and different tissues. By knocking down ELF4, the changes of H3K9me3 modification, DNA methylation and ZGA-related genes were analyzed. Our results showed that *ELF4* was expressed at all stages of early porcine embryos fertilized *in vitro* (IVF), with the highest expression level at the 8-cell stage. The embryonic developmental competency and blastocyst quality decreased after ELF4 knockdown (20.70% control vs. 17.49% si-scramble vs. 2.40% si-*ELF4*; *p* < 0.001). Knockdown of ELF4 induced DNA damage at the 4-cell stage. Interfering with ELF4 resulted in abnormal increases in H3K9me3 and DNA methylation levels at the 4-cell stage and inhibited the expression of genes related to ZGA. These results suggest that ELF4 affects ZGA and embryonic development competency in porcine embryos by maintaining genome integrity and regulating dynamic changes of H3K9me3 and DNA methylation, and correctly activating ZGA-related genes to promote epigenetic reprogramming. These results provide a theoretical basis for further studies on the regulatory mechanisms of ELF4 in porcine embryos.

## Introduction

E74 like ETS transcription factor 4 (ELF4), also known as myeloid elf-1-like factor (MEF), is a member of the E26 transformation-specific (ETS) transcription factor family and usually has transcriptional activation (1–4). Studies have found that that ELF4 promotes cell cycle progression from G1 to S phase and drives hematopoietic stem cell (HSC) development from stationary phase to G1 phase (5, 6). When the expression of ELF4 is silenced, the number of HSCs in the quiescent phase in the body will increase (6), and P53 enhances this effect of ELF4 (7). In cancer stem cells, regulation of the G1/S transition depends on the level of ELF4, a short-lived protein that regulates the cell cycle by being phosphorylated and ubiquitinated to control its expression level (8).

During embryonic development, certain DNA damage will be caused by various factors. It can be divided into two categories according to its source: endogenous DNA damage (copy errors, DNA base mismatches, and topoisomerase-DNA complexes) and exogenous DNA damage (ionizing radiation, ultraviolet radiation, exogenous chemical agent) (9). In response to this, cells activate their DNA damage repair system (10). Apoptotic programs initiate with excessively severe DNA damage (11). Excessive DNA damage can be detrimental to embryonic development. Before the S phase, the male and female pronuclei of embryos spontaneously generate large amounts of γH2AX during demethylation, and during the S phase, these DNA damages are repaired by activating PARP-1-related DNA repair mechanisms (12). However, it has been reported that in bovine embryos, DNA damage to sperm does not affect embryo division, but it can prevent embryo formation by inducing apoptosis (13). In mouse embryos, HT-2 toxin increased the formation of γH2AX, leading to increased DNA damage and impairing the development of early mouse embryos (14). It is reported that in a mouse model of enteritis, ELF4 regulates the expression of PARP-1 through its involvement in the DDR process, thereby inhibiting its oncogenic effects (15). Whether ELF4 has such a regulatory role in porcine preimplantation embryonic development is still unclear.

Embryos undergo a maternal-to-zygotic transition (MZT), a process known as ZGA, during which a number of embryonic genes are transcriptionally activated (16). In addition, ZGA is involved in multiple epigenetic modifications that are critical for preimplantation development (17). It is reported that abnormally high levels of H3K9me3 in mammalian somatic cell nuclear transfer (SCNT) embryos are an epigenetic barrier to reprogramming and the levels of histone demethylase KDM4D can regulate the levels of abnormal H3K9me3 in ZGA-stage embryos (18, 19). Greenberg et al. found an inverse relationship between promoter methylation and gene expression during mammalian preimplantation embryonic development, suggesting a suppressive effect of DNA methylation on embryonic development (20, 21). It is reported that the decreased expression level of ELF4 is due to the increased promoter methylation in a mouse model of colitis carcinoma and in humans traumatic stress disorder symptoms (15, 22). It is worth noting that cells open their chromatin structure and remove histone modifications at the site of DNA damage before DNA damage repair. If the removal is incomplete, epigenetic modifications will be abnormal (23). These indicate that there is a certain link between epigenetic modification, ELF4 and DNA damage response. However, the mechanism by which ELF4 is involved in regulating the development and epigenetic reprogramming of porcine IVF embryos remains unclear.

In this study, the expression of ELF4 in early porcine embryos and different tissues was detected. ELF4 was knocked down by cytoplasmic injection of specific siRNA, and the role of ELF4 in early embryo development was investigated by analyzing H3K9me3 modification, DNA methylation and ZGA-related gene changes. This study will provide a theoretical basis for further studying the mechanism of ELF4 on porcine embryonic development.

## Materials and methods

### Chemicals and animal ethics

All chemicals and culture media were purchased from Sigma-Aldrich (St. Louis, MO, USA) unless stated otherwise. The experimental protocol of this study was approved by the Animal Care and Use Committee of the First hospital of Jilin University (2019-099). All operations complied with the Chinese guidelines for the ethical treatment of experimental animals.

### Collection and *in vitro* maturation of porcine oocytes

Ovaries collected from the slaughterhouse were placed in a thermos with 0.9% NaCl containing 200 IU/ml of penicillin and streptomycin. Follicular fluid containing the cumulus-oocyte complex (COCs) was aspirated from follicles with a volume size of ~3–6 mm using a 10 ml syringe. The follicular fluid was placed in a 37°C incubator for about 15 min until a precipitate formed. The supernatant was discarded and COCs were washed twice with PBS containing 10% fetal bovine serum. The selected COCs were with at least three layers of cumulus cells. The COCs were placed to hormone-supplemented maturation solution (TCM-199 was used as base solution, 0.054 g d-glucose, 0.01 g sodium pyruvate, 0.0069 g cysteine, 0.3 g BSA, 1 ml penicillin/streptomycin (Gibco) and 10 ml follicular fluid were added to 100 ml base solution) at 38.5°C, 5% CO_2_ incubator for 22–24 h. Subsequently, they were transferred to hormone-free maturation medium and cultured for another 20 h, with a total of 42–44 h of mature culture. The oocytes discharged the first polar body (PB1) were considered mature and used for subsequent experiments.

### *In vitro* fertilization (IVF) and blastocyst quality grade assessment

Granulosa cells on the surface of COCs were digested with 2% hyaluronidase, and then mature oocytes were selected and placed in sperm capacitation fluid PGM (including 0.6312 g NaCl, 0.07456 g KCl, 0.00477 g KH_2_PO_4_, 0.00987 g MgSO_4_·7H_2_O, 0.2106 g NaHCO_3_, 0.0022 g sodium pyruvate, 0.3 g PVA, 0.077075 g calcium lactate, 0.04504 g theophylline, 0.002423 g cysteine, 0.01879 g d-glucose, 1 ml penicillin/streptomycin to 100 ml distilled water) for 30 min. Fresh semen was purchased from the pig farm of Jilin University. The solution was centrifuged by differential centrifugation (300 g, 20 min) by adding 2 ml of 90% Percoll (Solarbio, Beijing, China), 2 ml of 45% Percoll, and 2 ml of fresh semen. The supernatant was discarded, and the sperm was washed with 4 ml of DPBS containing 0.1% BSA and centrifuged at 300 g for 10 min. The supernatant was discarded and the pellet was resuspended with 1 ml of sperm capacitation solution PGM. The sperm were counted and the sperm concentration was adjusted to 2 × 10^5^ sperm/drop, and the sperm-oocytes were incubated in an incubator at 38.5°C and 5% CO_2_ for 5–6 h. The fertilized oocytes were washed and cultured in a PZM-3 medium (100 μl of PZM-3 per drop, 25 fertilized oocytes per drop).

In this paper, blastocyst quality grades were assessed according to the Gardner system. Gardner classifies blastocysts into six grades based on their expansion. Early blastocysts are grade 1, with blastocysts that are less than half the volume of the embryo. A blastocyst equal to or greater than half the volume of the embryo is a grade 2. A grade 3 blastocyst completely fills the embryo and is called a holoblast. a grade 4 blastocyst is characterized by a larger blastocyst volume than the early embryo and a thinner zona pellucida. When the trophectoderm begins to protrude through the zona pellucida, the blastocyst at this point is a grade 5 and is referred to as an incubating blastocyst. The hatching blastocyst is characterized by a blastocyst that has completely detached from the zona pellucida and is a grade 6 (24).

### siRNA synthesis and microinjection

The siRNA was designed and synthesized by Sangon Biotech, China. And the siRNA sequences were listed in Table 1. The siRNA concentration was all 20 μM. Microinjection of siRNA was performed using an Eppendorf Femtojet 4i microinjector (Eppendorf, Hamburg, Germany) after 4 h of sperm-oocytes co-incubation. After microinjection, fertilized oocytes were cultured in PZM-3 at 38.5°C and 5% CO_2_.

**Table 1:** The siRNA of ELF4 synthesis report form.

**Sample**	**Sequences (5'-3')**	
**(siRNA-ELF4)**	**Sense**	**Antisense**
si-677	GCUGGACGAUGUUCACAAUTT	AUUGUGAACAUCGUCCAGCTT
si-729	CCACUGAAGUCUUGCUCAATT	UUGAGCAAGACUUCAGUGGTT
si-scramble677	AUCAACGAUAUCCGGUUGGTT	CCAACCGGAUAUCGUUGAUTT
si-scramble729	AUUGCCACCGUAAUACGCUTT	AGCGUAUUACGGUGGCAAUTT

### RNA isolation and quantitative PCR (qPCR)

Total RNA was extracted from 50 to 100 porcine oocytes without zona pellucida and reverse transcribed into cDNA by the SuperScript TM IV Cells Direct TM cDNA Kit (Invitrogen, USA). RNA was extracted from various tissues of adult pigs by Trizol lysis buffer and reverse transcribed to cDNA using TransScript All-in-One-First-Strand cDNA (TranGen Biotech, Beijing, China). Primers used for qPCR were listed in Table 2. qPCR was performed using SYBR® Premix Ex Taq TM Reagent (TaKaRa). qPCR was a 20 μl reaction: 10 μl SYBR green premix, 2 μl primers, 2 μl cDNA, and 6 μl RNase-free water. The qPCR amplification conditions used a two-step method.

**Table 2:** Primers used in the qPCR analysis.

**Primer**	**Primer pair sequences (5**′**-3**′**)**
ACTB	F: CATCGTCCACCGCAAATG
	R: AGCCATGCCAATCTCATCT
ELF4	F: CTCGTATGTGCAGGGCGTGATG
	R: GGTTGGAAGTGGCTGAAGGTGAG
C-Myc	F: TACAACACCCGAGCGACAAC
	R: TCCGAAGGAAATCCAGCGTC
TDG	F: TCTGGAAGTGTCTGTTTATGTCGG
	R: CTGCTATTCGTGGCTGGTATT
KLF4	F: GAGGGAAGACCAGAATCCCTTGTA
	R: TAGAACCAAGACTCACCAAGCACC

### Immunofluorescence staining

Unless otherwise stated, all immunofluorescence (IF) staining of the embryos was performed at room temperature and the embryos were washed three times with PBS containing 0.1% polyvinylpyrrolidone (0.1% PVP/PBS) as a wash solution after each step of the staining process. Firstly, the zona pellucida on the surface of the embryo was dissolved with an Acid Tyrodes Solution (pH 2.5). Secondly, embryos were fixed with 4% paraformaldehyde in PBS for 30 min in the dark. Permeabilized with 2% Triton for 20 min. Thirdly, the embryos were blocked with 2% BSA at 37°C for 1 h. Then, blocked embryos were incubated overnight in primary antibodies at 4°C (Table 3) and then transferred to secondary antibodies for 1 h at 37°C (Table 3). Finally, embryonic nuclei were stained with 10 μg/ml 4',6-diamidino-2-phenylindole (DAPI) for 20 min. The 5 mC/5 hmC antibody was treated with 4N-HCl for 30 min after permeabilization and washing, followed by Tris-HCl (1 M, pH 7.6) for 30 min. Embryos are not washed before transfer from 4N-HCl to Tris-HCl. In addition, embryos stained with 5 mC/5 hmC antibodies did not need to be stained with DAPI.

**Table 3:** Antibodies used for Immunofluorescence staining and Western blotting.

**Antibody**	**Catalog** **No**.	**Manufacturer**	**Sources**	**Dilution** **ratio**
5mC	39649	Active Motif	Mouse	1:200
5hmC	39769	Active Motif	Rabbit	1:200
H3K9me3	ab8898	Abcam	Rabbit	1:200
γH2AX	2577	Cell signaling	Rabbit	1:200
α-Tubulin	AF7010	Affinity	Rabbit	1:1,000
ELF4	ab96075	Abcam	Rabbit	1:1,000
Goat anti- rabbit-HRP	SA00001-2	Proteintech	Goat	1:2,000
Goat anti-mouse IgG (Alexa Fluor^®^ 488)	A-11001	Invitrogen	Goat	1:500
Goat anti-rabbit IgG (Alexa Fluor^®^ 594)	A-11037	Invitrogen	Goat	1:500

### Western blotting

Total protein was extracted from 150 to 200 embryos using RIPA Lysis Buffer (Solarbio, Beijing, China). The protein samples were separated by Biofuraw TM Precast Gel (Tanon, Shanghai, China), and the electrophoresis condition was 120 V, 60 min. Subsequently, the protein bands were transferred to the Immobilon-p transfer membrane (Millipore, MA, USA), and the transfer condition was 300 mA, 60 min. Blocking was performed with 5% nonfat dry milk in PBST. The membrane was first incubated with primary antibodies (Table 3) overnight at 4°C. It was then incubated with horseradish peroxidase (HRP)-conjugated secondary antibody (Table 3) for 1 h at room temperature. After incubation with primary antibody and secondary antibody, the membrane was washed three times with PBST, respectively. Then the protein bands were visualized using a Tanon 5200 automated fluorescence/chemiluminescence imaging analysis system (Tanon, Shanghai, China).

### Microscopic observation and image analysis

The protein fluorescence in the images was detected by fluorescence microscopy (Nikon, Tokyo, Japan) at the same exposure time. Next, the total fluorescence intensity of each image was evaluated by ImageJ software (National Institutes of Health, Bethesda, MD) under the same parameter conditions.

### Statistical analysis

Each experiment was biologically replicated at least three times. Data were analyzed by GraphPad Prism8 software (GraphPad Software, San Diego, America), using unpaired samples *T*-test or ordinary one-way ANOVA, *p* < 0.05 was considered statistically significant; *p* < 0.01 was considered extremely significant.

## Results

### Expression analysis of ELF4 in early porcine embryos and different tissues

**Figure 1:**
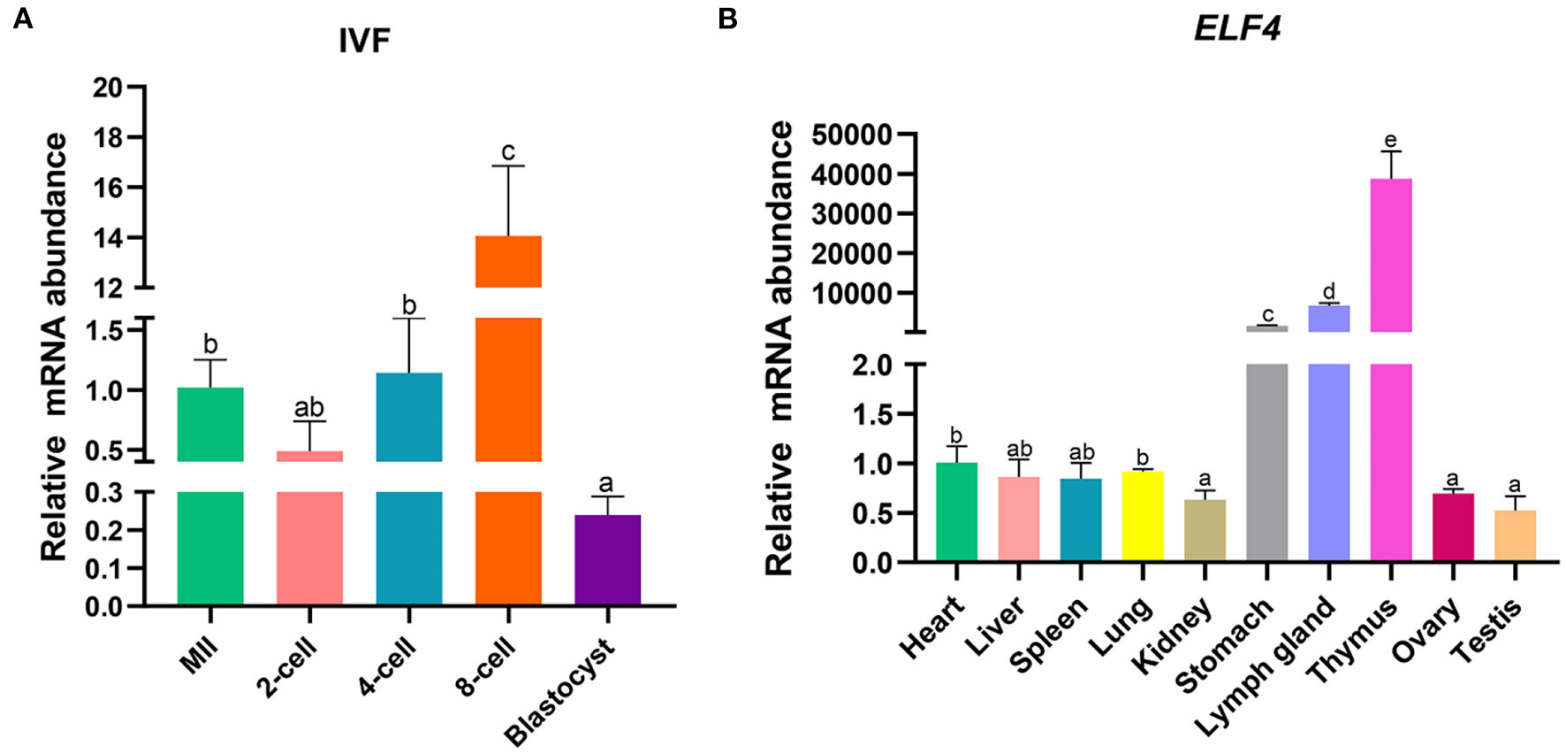
Expression profile of *ELF4* in pigs. **(A)** Expression levels of *ELF4* in early IVF embryos. **(B)** Expression analysis of *ELF4* in different tissues. Different lowercase letters indicate statistical differences (*p* < 0.05).

To explore the effect of ELF4 on the preimplantation development of porcine IVF embryos, we first examined its expression changes at different stages of early embryonic development. The results showed that *ELF4* was expressed at all stages of early embryos. Compared with the MII oocytes stage, the expression level of *ELF4* was significantly increased in the 8-cell stage and significantly decreased in the blastocyst stage (Figure 1A). Next, we examined the expression level of ELF4 in porcine different tissues. The relative abundance of *ELF4* in different tissues was detected by qPCR. *ELF4* was highly expressed in thymus, lymph gland and stomach, with the highest expression in the thymus (*p* < 0.001). However, the expression levels were lower in kidney, ovary and testis, and no significant difference (*p* > 0.05) was observed among them (Figure 1B).

### Screening of interfering RNAs of ELF4

**Figure 2:**
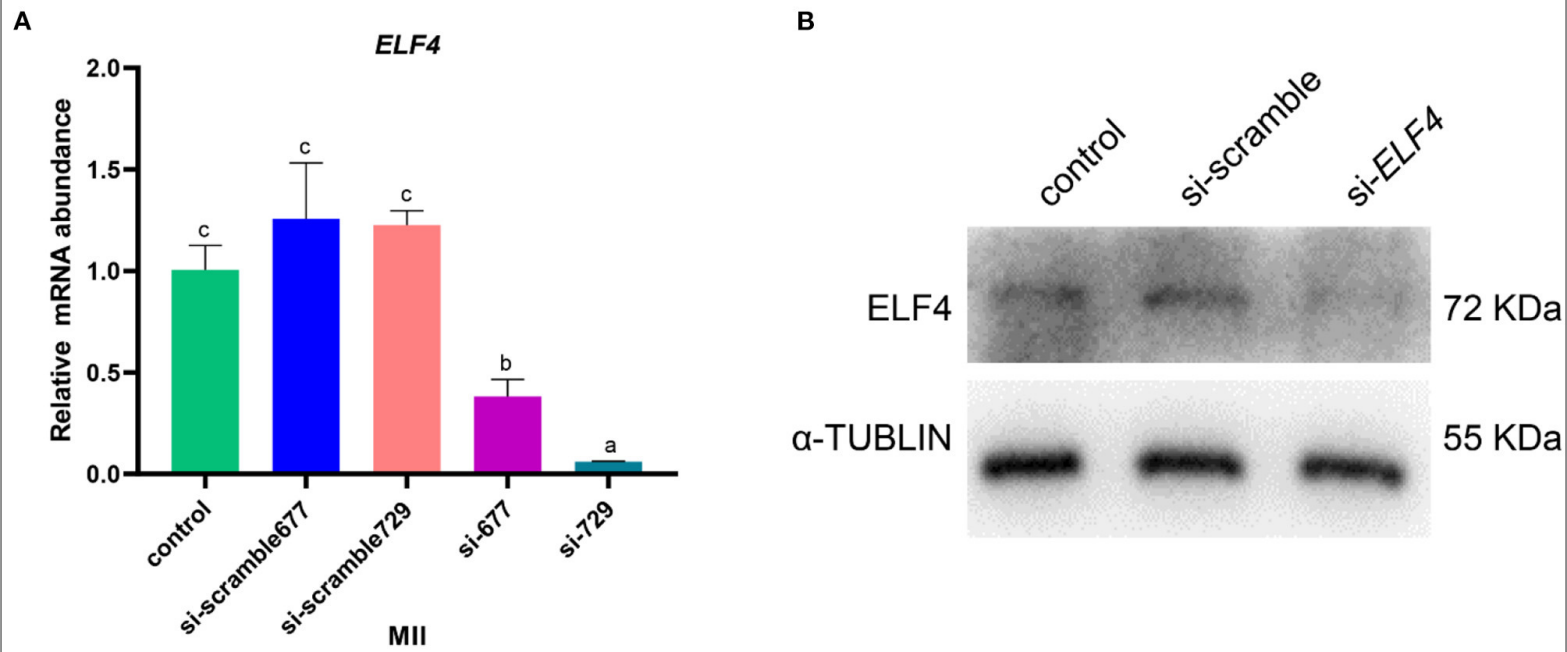
Screening of interfering RNAs of ELF4. **(A,B)** Interference efficiency of ELF4 siRNA at the transcriptional **(A)** and protein levels **(B)**. Different lowercase letters indicate statistical differences (*p* < 0.05).

We designed targeting siRNAs for the 677th and 729th positions of the ELF4 coding sequence, respectively, named as si-677 and si-729. We also designed the corresponding scramble RNA, named as si-scramble 677 and si-scramble729, respectively. The scramble RNA served as the negative control of siRNA. The interference efficiency of siRNA was detected by real-time PCR. The results showed that both siRNAs significantly decreased the expression level of *ELF4* (Figure 2A). The interference efficiency of si-729 was significantly reduced by 90% (*p* < 0.001), and the interference efficiency of si-677 was 60% (*p* < 0.01). Therefore, we used si-729 and si-scramble729 in subsequent experiments. For the convenience of labeling, we labeled si-scramble729 as si-scramble in subsequent experiments. Next, we examined the interference efficiency of si-729 at the protein level. Results showed that protein levels of ELF4 were reduced compared with those in the control group (Figure 2B).

### Knockdown of ELF4 was detrimental to porcine blastocyst formation and impaired blastocyst quality

**Figure 3:**
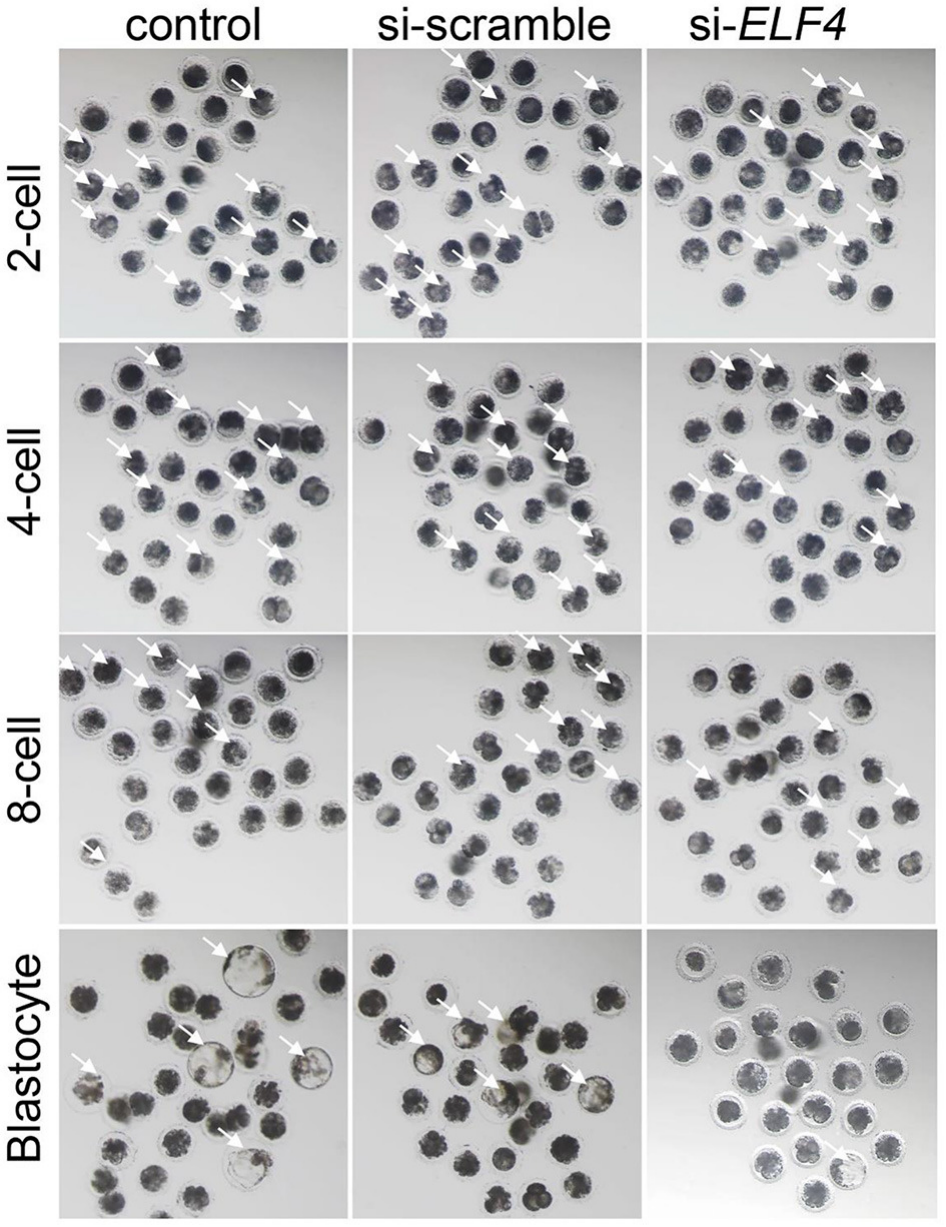
The knockdown of ELF4 is not conducive to the development of porcine IVF embryos. Bright-field phenotypes of early embryonic development.

**Figure 4:**
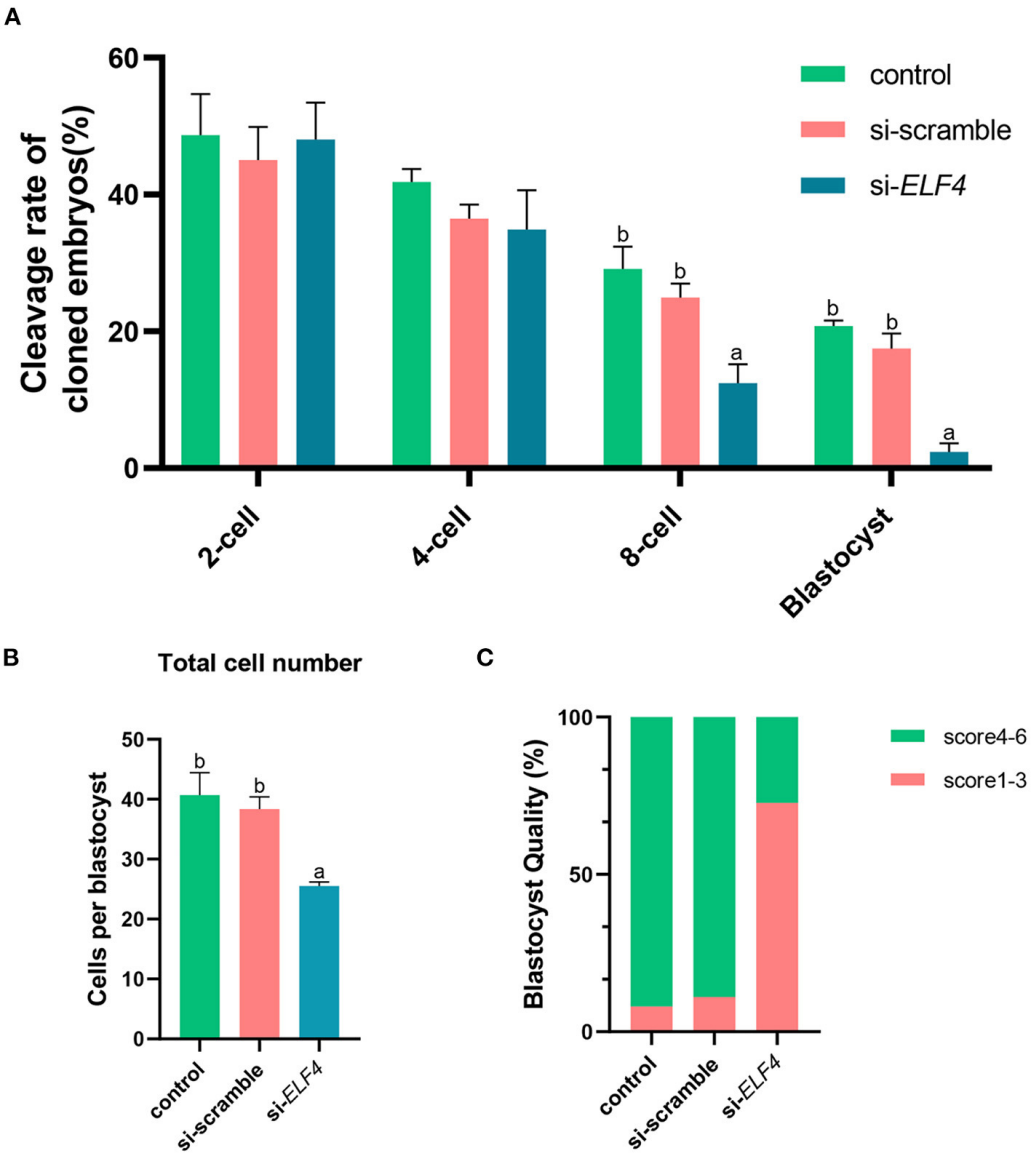
Knockdown of ELF4 reduced blastocyst development rate and blastocyst quality in porcine IVF embryos. **(A)** The embryonic development capacity of control, si-scramble and si-*ELF4* groups. **(B,C)** Blastocyst quality inspection: blastocyst cell count **(B)**, blastocyst grade **(C)**. Different lowercase letters indicate statistical differences (*p* < 0.05).

We defined the group injecting with scramble as si-scramble, defined the group injecting with ELF4 siRNA as si-*ELF4*, and defined the untreated group as the control group. The siRNA was injected into embryos 4 h after IVF. Compared with the 2-cell and 4-cell groups, the 8-cell rate (29.15% control vs. 24.92% si-scramble vs. 12.40% si-*ELF4*; *p* < 0.01) and blastocyst rate (20.70% control vs. 17.49% si-scramble vs. 2.40% si-*ELF4*; *p* < 0.001) in the si-*ELF4* group was significantly lower (Figures 3, 4A). Then we evaluated the blastocyst quality. The blastocyst cell number of si-*ELF4* was significantly reduced (40.67 ± 3.09 control vs. 38.33 ± 1.70 si-scramble vs. 25.50 ± 0.50 si-*ELF4*; *p* < 0.01; Figure 4B) when compared with the other two groups. The blastocyst grade analysis showed that about 27% of si-*ELF4* blastocysts were grades 4–6, and about 73% were grades 1–3. There was no difference in the quality between the control group and si-scramble group blastocysts, about 90% for grades 4–6 and about 10% for grades 1–3 (Figure 4C). The above results showed that the blastocyst rate and blastocyst quality of embryos decreased by interfering with ELF4.

### Interference with ELF4 induced DNA damage in the 4-cell embryos

**Figure 5:**
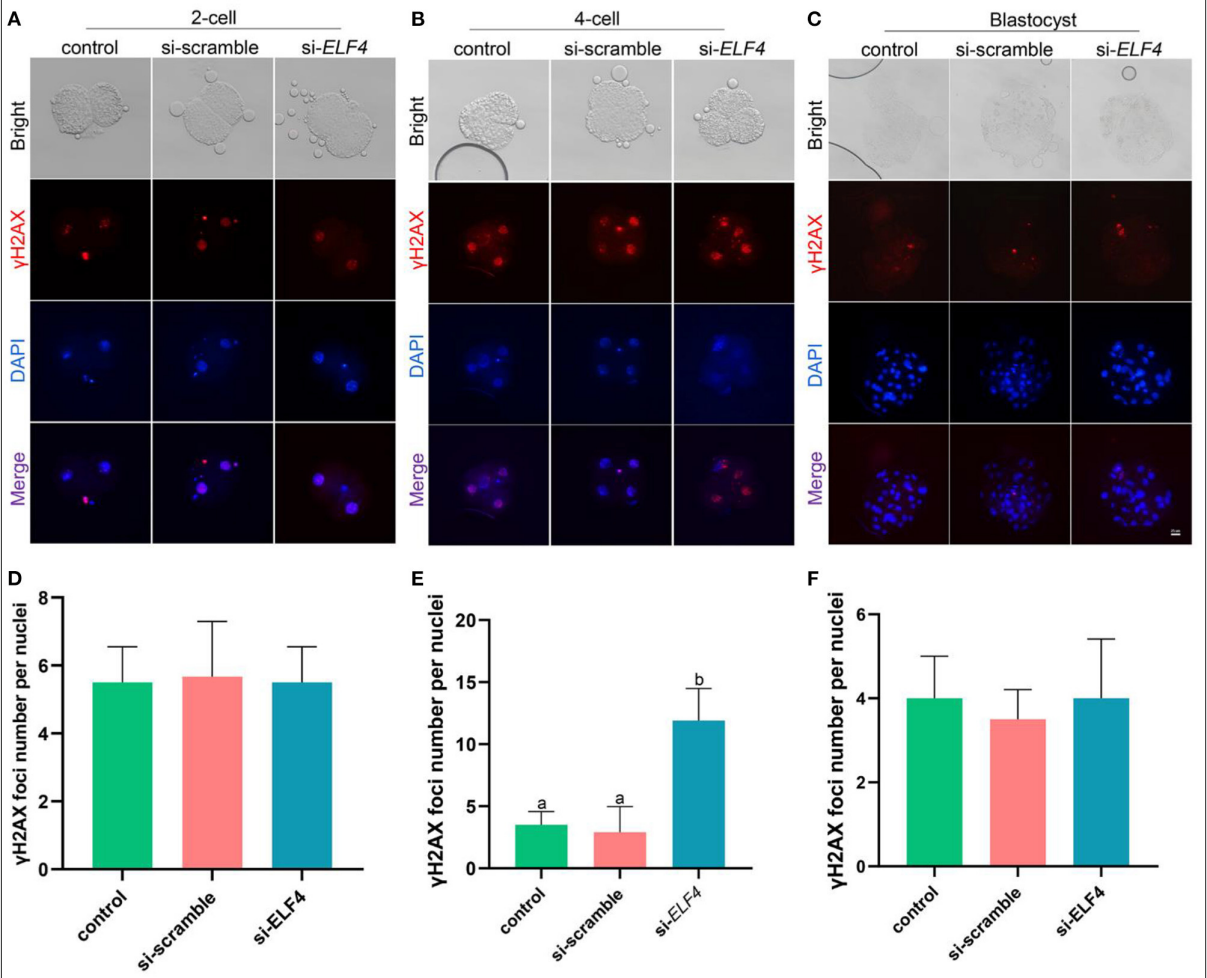
Effects of ELF4 on DNA damage during preimplantation development of porcine IVF embryos. **(A–C)** Immunofluorescence staining of γH2AX foci in the control, si-scramble, and si-*ELF4* embryos at the 2-cell, 4-cell and blastocyst stages. Scale bars represented 25 μm. **(D–F)** Semi-quantitative fluorescence intensity analysis of the γH2AX staining at the 2-cell, 4-cell and blastocyst stages. Different lowercase letters indicate statistical differences (*p* < 0.05).

To identify the endogenous DNA damage, we examined γH2AX foci formation. The results showed that γH2AX lesions were significantly higher in the si-*ELF4* group than that in controls (3.5 ± 1.02 control vs. 2.91 ± 1.98 si-scramble vs. 11.91 ± 2.47 si-*ELF4*; *p* < 0.001; Figures 5B,E) at the 4-cell stage. However, the γH2AX foci were not statistically different between groups at 2-cell (Figures 5A,D) and blastocyst stages (Figures 5C,F).

### Knockdown of ELF4 increased H3K9me3 levels at the 4-cell stage of porcine embryos

**Figure 6:**
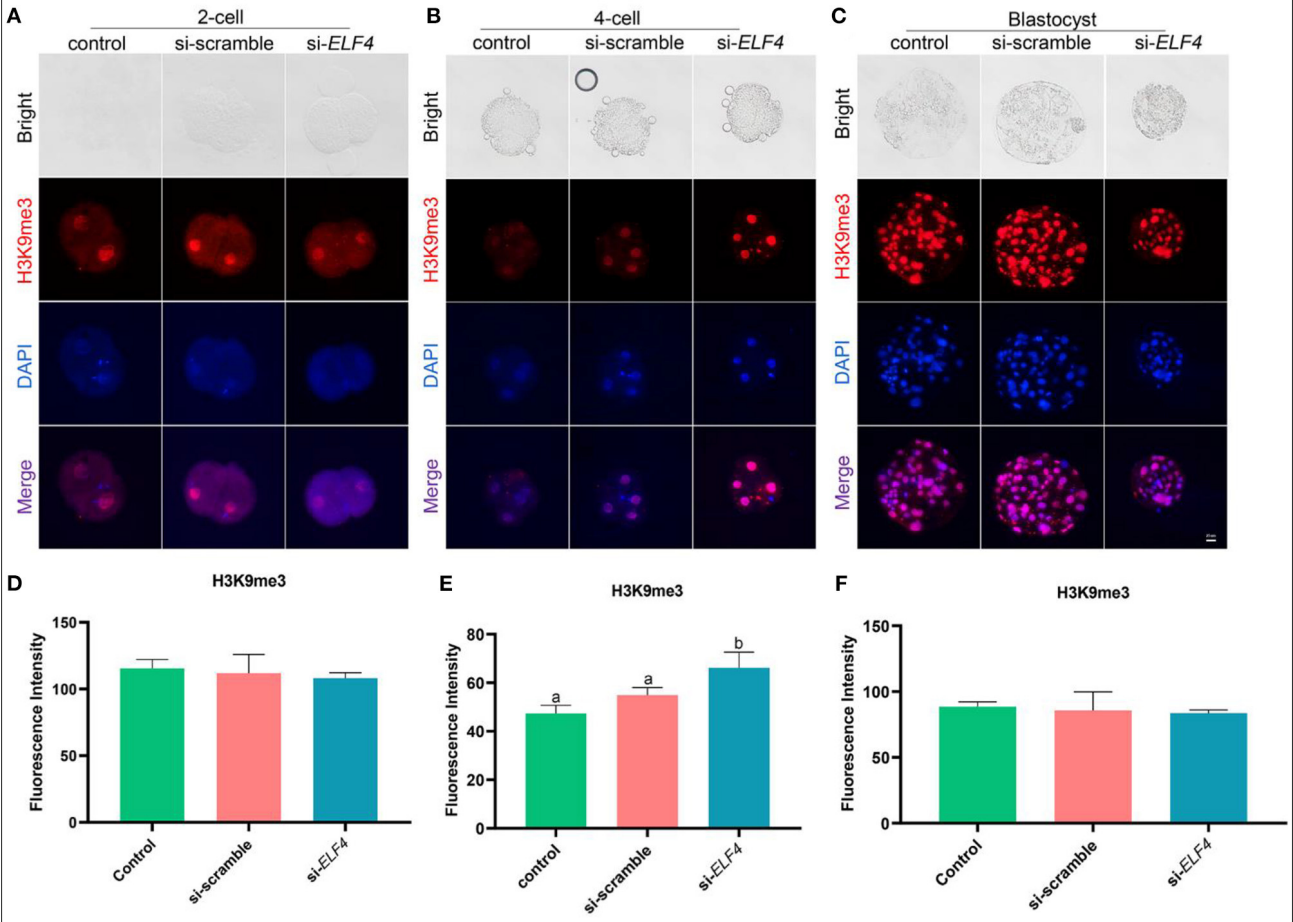
Dynamic changes of H3K9me3 at different stages of preimplantation IVF development after ELF4 knockdown. **(A–C)** Immunofluorescence staining of H3K9me3 in the control, si-scramble, and si-*ELF4* embryos. Scale bars represented 25 μm. **(D–F)** Semi-quantitative fluorescence intensity analysis of the H3K9me3 staining. Different lowercase letters indicate statistical differences (*p* < 0.05).

We examined the dynamic changes of H3K9me3 levels in each group, the results showed that the level of H3K9me3 increased in the si-*ELF4* group at the 4-cell stage (47.33 ± 2.74 control vs. 54.87±2.61 si-scramble vs. 66.13 ± 5.29 si-*ELF4*; *p* < 0.01; Figures 6B,E). However, at the 2-cell (Figures 6A,D) and blastocyst stages (Figures 6C,F), there was no statistical difference in H3K9me3 levels among the three groups.

### Knockdown of ELF4 elevated genome-wide DNA methylation at the 4-cell stage of porcine embryos

**Figure 7:**
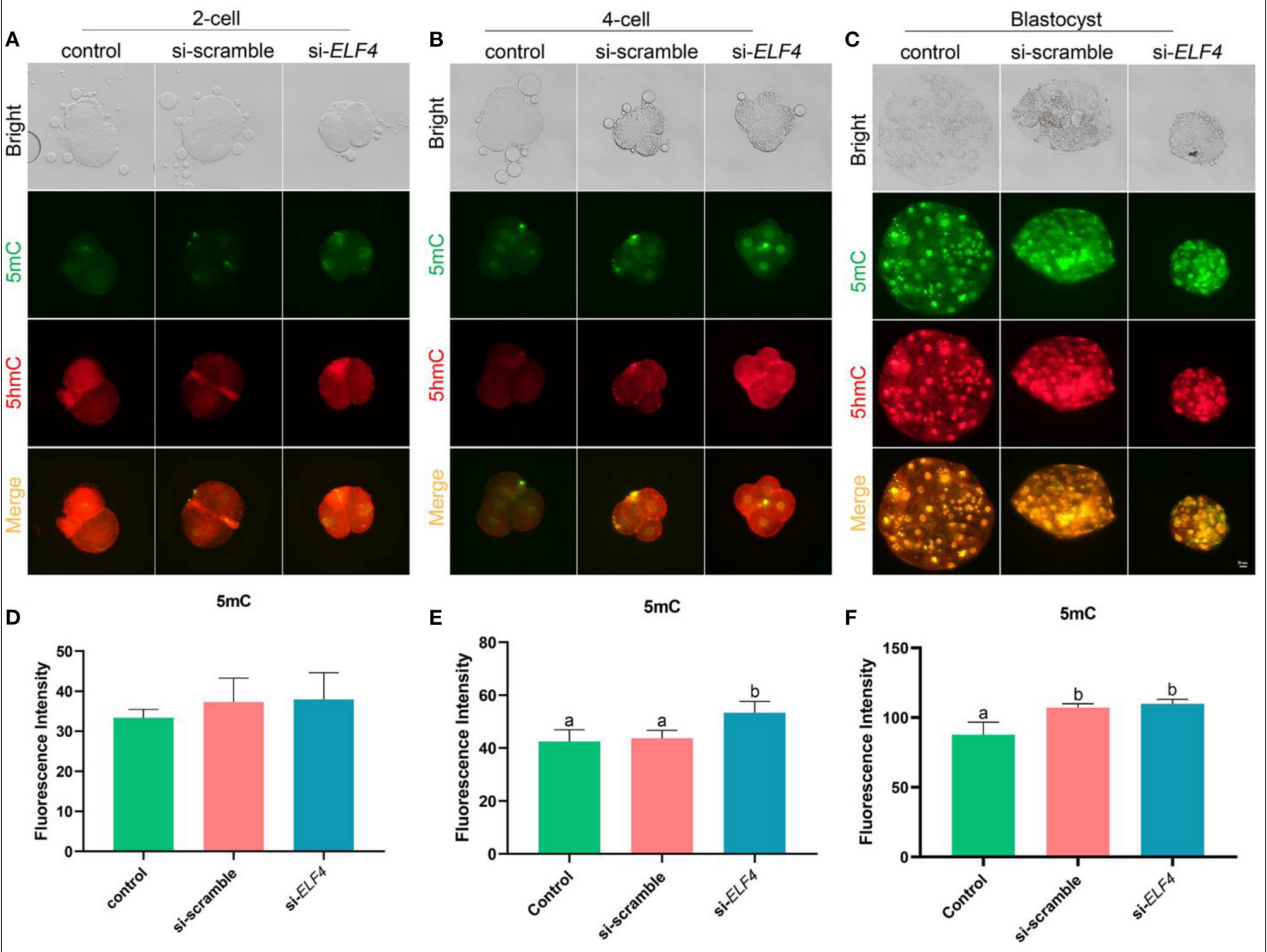
Dynamic changes of DNA methylation at different stages of the preimplantation embryo after ELF4 knockdown. **(A–C)** Immunofluorescence staining of DNA methylation in the control, si-scramble, and si-*ELF4* embryos. Scale bars represented 25 μm. **(D–F)** Semi-quantitative fluorescence intensity analysis of the DNA methylation staining. Different lowercase letters indicate statistical differences (*p* < 0.05).

We investigated changes in DNA methylation levels after the knockdown of ELF4. The results showed the DNA methylation level at the 4-cell stage was significantly higher in the si-ELF4 group compared to the other two groups (42.52 ± 3.60 control vs. 43.68 ± 2.46 si-scramble vs. 53.37 ± 3.45 si-*ELF4*; *p* < 0.01; Figures 7B,E). DNA methylation levels were not different between the three groups at the 2-cell stage (Figures 7A,D). At the blastocyst stage, the DNA methylation level of si-*ELF4* was higher than that of the control group but not remarkably different from that of the si-scramble group (Figures 7C,F).

### ELF4 affected the expression of porcine ZGA-related genes

**Figure 8:**
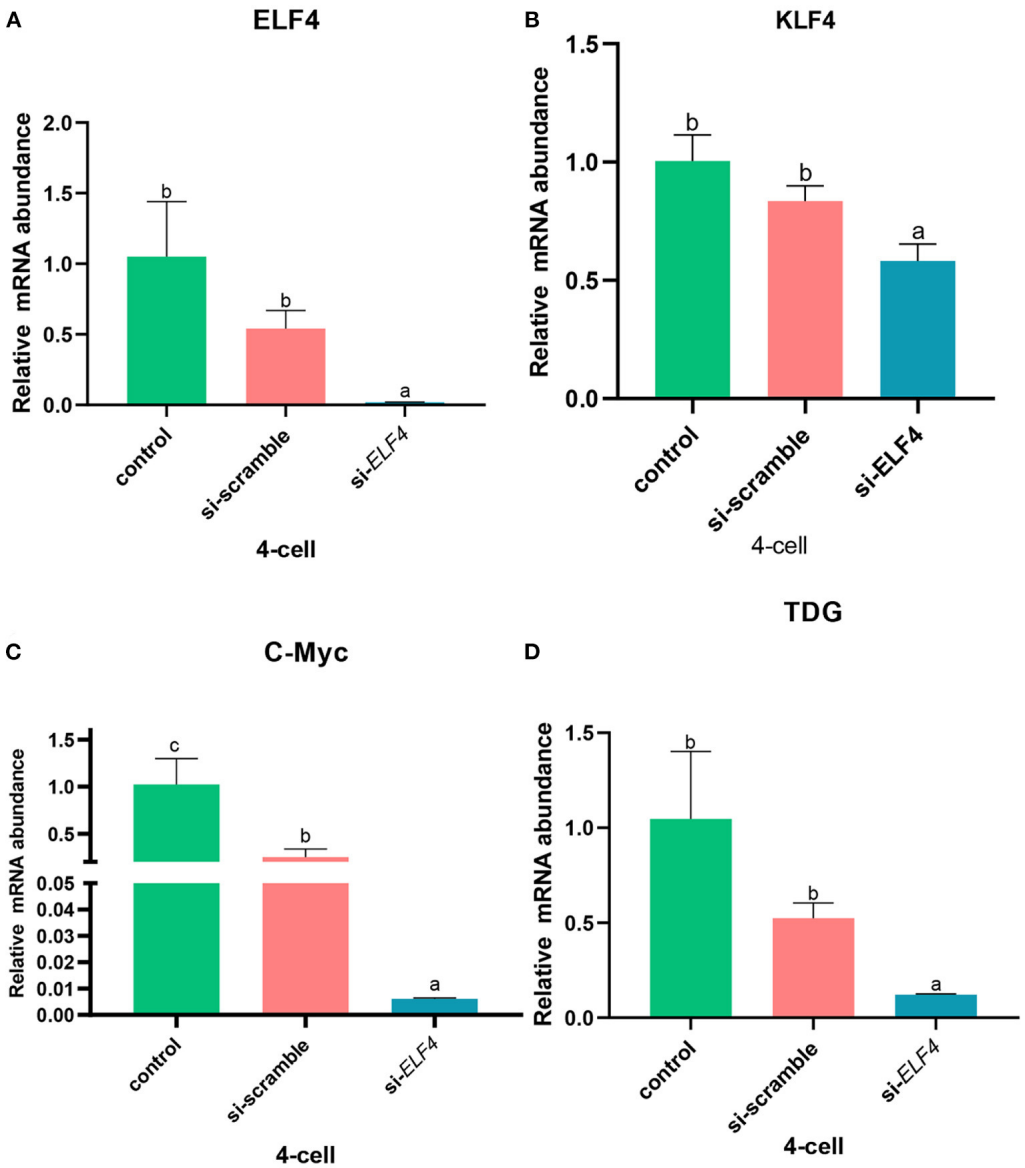
Knockdown of ELF4 affected ZGA-related genes initiation in porcine embryos. **(A)** Transcriptional levels of *ELF4*. **(B–D)** Transcriptional levels of ZGA-related genes *KLF4* **(B)**, *C-Myc* **(C)** and *TDG* **(D)** in the control, si-scramble and si-*ELF4* embryos. Different lowercase letters indicate statistical differences (*p* < 0.05).

Next, we examined several key ZGA-related genes in the porcine embryo at the 4-cell stage. After knockdown of ELF4, the expression levels of genes positively correlated with ZGA decreased (Figure 8). Compared with si-scramble, the transcription level of *ELF4* was significantly reduced (Figure 8A; *p* < 0.01). The transcription level of *KLF4, C-Myc* and *TDG* were remarkably down-regulated by1.5 times (Figure 8B; *p* < 0.05), 14 times (Figure 8C; *p* < 0.001) and 5 times (Figure 8D; *p* < 0.001), respectively.

## Discussion

In recent years, researchers have found that the ETS family is involved in various physiological processes during embryonic development, which regulates blood vessel development (25), immune cell generation and differentiation (26), epithelial cell differentiation (27), etc. ELF4 is a member of the ETS transcription factor family (4). Hedvat et al. (3) have found that ELF4 regulates the immune response and the development of immune-related cells, and plays an important role in hematopoiesis. In addition, ELF4 is also involved in the regulation of the cell cycle (5), mediates the differentiation of adipogenic osteoblasts (4), and participates in DNA damage repair (15). However, the role of ELF4 in the regulation of embryonic development and epigenetic reprogramming remains unclear.

DNA damage can disrupt metabolic homeostasis and trigger inflammatory responses (28). Moreover, DNA damage can also disrupt early blastocyst formation, which is detrimental to later embryonic development (14, 29, 30). ELF4 plays an important regulatory role in the DNA damage response. It was found that γH2AX foci in Elf4-KO cells were reduced after γ-irradiation, leading to a decrease in apoptosis involved of p53 (31). In a study of a model of enteritis, ELF4 deficiency led to more severe DNA damage in mice *in vitro* and *in vivo*, and ELF4 deficiency made host cells more prone to cancer (15). However, the DNA damage response regulatory mechanism of ELF4 has not been reported in the early embryonic development in pigs. Our results showed that knockdown of ELF4 increased DNA damage in 4-cell embryos, which indicated that ELF4 affected early embryonic development in pigs by controlling genome integrity.

H3K9me3 and DNA methylation are important epigenetic modifications with important regulatory roles in embryonic development. The abnormal dynamic changes of H3K9me3 can inhibit the expression of pluripotency genes, thereby inhibiting the proliferation and differentiation of mouse embryonic stem cells (32). It is reported that abnormal DNA methylation can also inhibit the differentiation of human embryonic stem cells by inhibiting the expression of pluripotency genes (33). In addition, H3K9me3 and DNA methylation modification levels are also associated with DNA damage repair in embryonic development, and their abnormal elevation can increase DNA damage in embryonic development and inhibit the expression of ZGA genes (18, 34, 35). But it is unclear whether ELF4 regulates epigenetic modifications in porcine embryonic development. Our results showed that knockdown of ELF4 resulted in abnormal epigenetic reprogramming in porcine 4-cell embryos, mainly manifested by abnormally elevated levels of H3K9me3 and DNA methylation. These results indicated that ELF4 helps to regulate the normal dynamic changes of H3K9me3 and DNA methylation modification, which is conducive to epigenetic reprogramming.

Embryos undergo ZGA during early development, and its initiation begins with the expression of genes involved in ZGA. The expression of genes related to ZGA is regulated by some transcription factors, and their abnormal expression can hinder embryonic development. Wang et al. (36) found that Klf4, Nr5a2 and Rarg exhibited high transcriptional activity and expression levels in goat 2-cell embryos, and these transcription factors were positively correlated with ZGA-related genes. It was reported that knockdown of Dux resulted in major defects in embryo differentiation and a significant reduction in blastocyst development rate during early embryonic development in mice *in vitro* fertilized embryos, while transcript levels of ZGA genes, such as *MERVL, Zscan4, Sp110*, and *Eif1a* decreased (37). However, whether and how *ELF4* regulates ZGA genes has not been reported yet. Our results showed that knockdown of *ELF4* resulted in decreased transcript levels of ZGA-related genes. These results demonstrated that the down-regulation of ELF4 was detrimental to the activation process of the porcine ZGA genes and consequently to the normal development of the subsequent blastocyst.

In conclusion, our results indicate that ELF4 affects ZGA and embryonic development competency in porcine embryos by maintaining genome integrity and regulating dynamic changes of H3K9me3 and DNA methylation, and correctly activating ZGA-related genes to promote epigenetic reprogramming. Our findings firstly describe the function of ELF4 in early porcine embryonic development, and provide a theoretical basis for further studies of the regulation mechanism of ELF4 in porcine embryos.

## Data availability statement

The original contributions presented in the study are included in the article/supplementary material, further inquiries can be directed to the corresponding authors.

## Ethics statement

The animal study was reviewed and approved by First Hospital, Jilin University.

## Author contributions

ZL conceived the project and supervised the experiments. ZL and HY revised the manuscript. LS and YaZ performed most of the experiments, analyzed data, and wrote the manuscript. YuZ, XK, and DZ participated in the part of experiments and data analysis. All authors read and approved the final manuscript.

## Funding

This research was supported by the National Natural Science Foundation of China (No. 31972874), the National Key R&D Program of China (No. 2017YFA0104400), and the Program for Changjiang Scholars and Innovative Research Team in University (No. IRT_16R32).

## Conflict of interest

The authors declare that the research was conducted in the absence of any commercial or financial relationships that could be construed as a potential conflict of interest.

## Publisher's note

All claims expressed in this article are solely those of the authors and do not necessarily represent those of their affiliated organizations, or those of the publisher, the editors and the reviewers. Any product that may be evaluated in this article, or claim that may be made by its manufacturer, is not guaranteed or endorsed by the publisher.
